# Detection of *Naja atra* Cardiotoxin Using Adenosine-Based Molecular Beacon

**DOI:** 10.3390/toxins9010024

**Published:** 2017-01-07

**Authors:** Yi-Jun Shi, Ying-Jung Chen, Wan-Ping Hu, Long-Sen Chang

**Affiliations:** 1 Institute of Biomedical Sciences, National Sun Yat-Sen University, Kaohsiung 804, Taiwan; a786514@gmail.com (Y.-J.S.); yjchen@mail.nsysu.edu.tw (Y.-J.C.); 2 Department of Biotechnology, Kaohsiung Medical University, Kaohsiung 807, Taiwan; wphu@cc.kmu.edu.tw

**Keywords:** snake venom, cardiotoxin, molecular beacon, polyadenosine, coralyne

## Abstract

This study presents an adenosine (A)-based molecular beacon (MB) for selective detection of *Naja atra* cardiotoxin (CTX) that functions by utilizing the competitive binding between CTX and the poly(A) stem of MB to coralyne. The 5′- and 3′-end of MB were labeled with a reporter fluorophore and a non-fluorescent quencher, respectively. Coralyne induced formation of the stem-loop MB structure through A_2_-coralyne-A_2_ coordination, causing fluorescence signal turn-off due to fluorescence resonance energy transfer between the fluorophore and quencher. CTX3 could bind to coralyne. Moreover, CTX3 alone induced the folding of MB structure and quenching of MB fluorescence. Unlike that of snake venom α-neurotoxins, the fluorescence signal of coralyne-MB complexes produced a bell-shaped concentration-dependent curve in the presence of CTX3 and CTX isotoxins; a turn-on fluorescence signal was noted when CTX concentration was ≤80 nM, while a turn-off fluorescence signal was noted with a further increase in toxin concentrations. The fluorescence signal of coralyne-MB complexes yielded a bell-shaped curve in response to varying concentrations of *N. atra* crude venom but not those of *Bungarus multicinctus* and *Protobothrops mucrosquamatus* venoms. Moreover, *N. nigricollis* venom also functioned as *N. atra* venom to yield a bell-shaped concentration-dependent curve of MB fluorescence signal, again supporting that the hairpin-shaped MB could detect crude venoms containing CTXs. Taken together, our data validate that a platform composed of coralyne-induced stem-loop MB structure selectively detects CTXs.

## 1. Introduction

Molecular beacons (MBs) have been widely used as an analytical platform for detecting small biological molecules [1,2]. The fundamental structure of MB is a short oligonucleotide chain with a single-stranded loop and a hybridized stem structure. By attaching a fluorophore and a quencher to the ends of the beacon strand, the fluorescence is strongly quenched due to the fluorescence resonance energy transfer (FRET) from the fluorophore to the quencher, owing to their close proximity following stem formation. Therefore, the on-off fluorescence system could rapidly detect molecules interacting with MB [1,2].

Snake venom contains a number of pharmacologically active proteins. Although enzyme-linked immunoassay has long been employed for detection of snake venom proteins, it could not rapidly detect the proteins due to time-consuming procedures [3]. If snake venom proteins could induce ‘turn-on’ or ‘turn-off’ fluorescence signal transduction of MBs, the beacons might be employed for rapid detection of snake venom proteins. Previous studies showed that *Naja atra* cardiotoxin 2 (CTX2) could bind with ATP and dATP [4], suggesting the possibility that CTXs may interact with adenosine (A)-rich oligonucleotide chain. Additionally, it has been proven that coralyne could intercalate strongly to poly(A) or poly(dA) and drive the formation A_2_-coralyne-A_2_ coordination [5]. Consistent with this finding, Kuo and Tseng [6] reported that coralyne promotes the conformational change from an extended structure to a hairpin structure in a DNA probe with the nucleotide sequence A_12_-CATCATAGTCGAGTGTCCAGGG-A_12_. The binding of coralyne with the DNA probe functionalized with a reporter of carboxyfluorescein (FAM) at the 5′-end and a quencher of 4-([4-(dimethylamino)phenyl]azo)-benzoic acid (DABCYL) at the 3′-end of A_12_-MB-A_12_ leads to quenching of FAM fluorescence because the stem hydride brings FAM and DABCYL to a close proximity [6]. If CTXs could compete with coralyne for binding with the A_12_ nucleotide segment in the MB DNA probe, separation of FAM and DABCYL units might restore FAM fluorescence. To test this hypothesis, the present study investigated the effect of CTXs on the FRET signal of the MB DNA probe. Our data showed that CTXs could bind with both coralyne and A_12_-MB-A_12_, and in their presences, the fluorescence signal from coralyne-MB complexes yielded bell-shaped concentration-dependent curves. Moreover, coralyne-based stem-loop MB structure was found to specifically discriminate CTXs from α-neurotoxins in snake venom.

## 2. Results and Discussion

**Figure 1:**
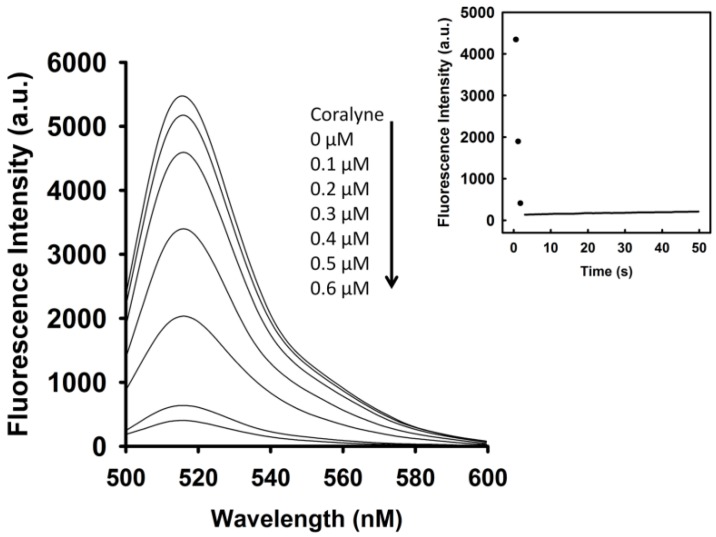
Fluorescence intensity at 520 nm of A_12_-MB-A_12_ was reduced by titrating with coralyne. FAM/DABCYL-labeled A_12_-MB-A_12_ (10 nM) was titrated with increasing coralyne concentrations as indicated. (**Inset**) Time course measurement of FAM intensity (520 nm) of 10 nM A_12_-MB-A_12_ upon the addition of 0.6 μM coralyne.

To identify the optimal concentration of coralyne for maximal quenching of the fluorescence between FAM and DABCYL, a 10 nM A_12_-MB-A_12_ DNA probe was titrated with coralyne. As shown in Figure 1, the fluorescence intensity of FAM was reduced by addition of coralyne, and it reduced sharply within 10 s at 520 nm. The fluorescence quenching reached a saturation level after addition of 0.6 μM coralyne, indicating that the hairpin structure of A_12_-MB-A_12_ was more stable at coralyne concentrations exceeding 0.6 μM. According to the change in the FAM fluorescence intensity, the binding affinity of coralyne towards A_12_-MB-A_12_ was calculated. The dissociation constant (K_d_) of coralyne for A_12_-MB-A_12_ was 0.5 μM. This is in agreement with the finding that coralyne binds to poly(A) with an association constant of 1.8 × 10^6^ M^−1^ at pH 7.0 [7,8].

**Figure 2:**
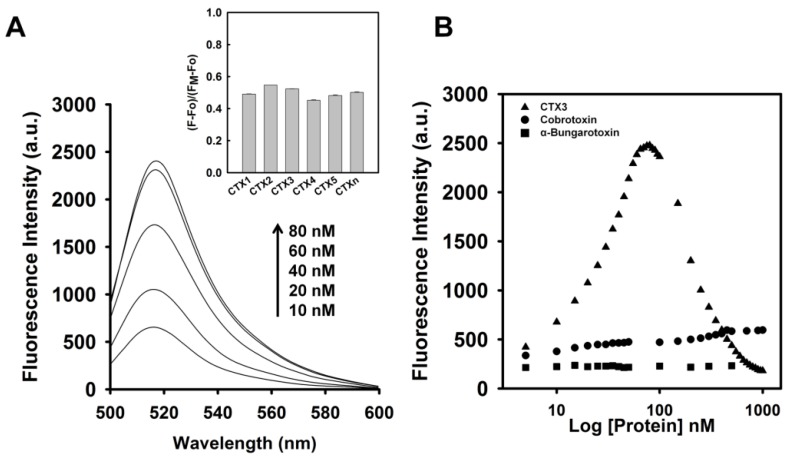
Effect of CTX3 on fluorescence intensity at 520 nm of hairpin-shaped A_12_-MB-A_12_. (**A**) Fluorescence spectra of solutions of 10 nM FAM/DABCYL-labeled A_12_-MB-A_12_ and 0.6 μM coralyne in the presence of indicated CTX3 concentrations. (**Inset**) Relative fluorescence intensity of solutions of 10 nM FAM/DABCYL-labeled A_12_-MB-A_12_ and 0.6 μM coralyne in the presence of 80 nM CTXs. Fo and F corresponded to fluorescence intensity at 520 nm of FAM as measured from MB probe in the absence or presence of toxin molecules. F_M_ represented the fluorescence intensity at 520 nm of FAM as measured from the solution of 10 nM A_12_-MB-A_12_ in the absence of coralyne; (**B**) Effect of CTX3, cobrotoxin, and α-bungarotoxin on fluorescence intensity at 520 nm of a solution containing 10 nM FAM/DABCYL-labeled A_12_-MB-A_12_ and 0.6 μM coralyne. The hairpin-shaped MB was titrated with indicated concentrations of CTX3, cobrotoxin, and α-bungarotoxin.

To assess the concentration-dependent effect of CTX3 on the FAM fluorescence of hairpin-shaped MB, a solution containing 10 nM MB and 0.6 μM coralyne was titrated with CTX3. As shown in Figure 2A, maximum restoration of FAM fluorescence was noted with the addition of 80 nM CTX3. The fluorescence turn-on assay required 10 s to achieve maximum recovery of FAM fluorescence (Figure S1, Supplementary Materials). The fitted curve was used for quantification of CTX3 with a correlation coefficient of 0.995, and the detection limit of 0.03 nM could be reached based on the definition of three times the deviation of the blank signal (3σ). Noticeably, CTX3 caused a recovery of FAM fluorescence to approximately 50% of that noted with coralyne-free MB. Restoration of FAM fluorescence increased with increasing the CTX3 concentration up to 80 nM (Figure 2B). Likewise, the addition of 80 nM CTX1, CTX2, CTX4, CTX5, and CTXn restored the FAM fluorescence of hairpin-shaped MB (Inset of Figure 2A). To test the specificity of hairpin-shaped MB in detecting CTXs, the effect of cobrotoxin (a short-chain α-neurotoxin from *N. atra* venom) and α-bungarotoxin (a long-chain α-neurotoxin from *Bungarus multicinctus* venom) on FAM fluorescence was analyzed. CTXs, cobrotoxin, and α-bungarotoxin structurally adopt a three-loop folding topology [9,10,11,12], but CTXs and α-neurotoxins show distinct pharmacological activities [13]. Moreover, genetic analyses have proven that CTXs, cobrotoxin, and α-bungarotoxin share a common gene structure and originate from the same ancestor [13,14,15,16,17]. As shown in Figure 2B, cobrotoxin and α-bungarotoxin did not notably restore the FAM fluorescence of hairpin-shaped MB at test concentrations up to 1000 nM. These results indicated that hairpin-shaped MB was more selective for detecting CTXs over α-neurotoxin. Nevertheless, a notable reduction in the FAM fluorescence of A_12_-MB-A_12_ was noted when the concentration of added CTX3 was >100 nM, and CTX3 quenched the fluorescence signal of A_12_-MB-A_12_ in a concentration-dependent manner. Likewise, titration of coralyne-A_12_-MB-A_12_ complexes with other CTX isotoxins also yielded bell-shaped curves of FAM fluorescence signal (Figure S2, Supplementary Materials).

**Figure 3:**
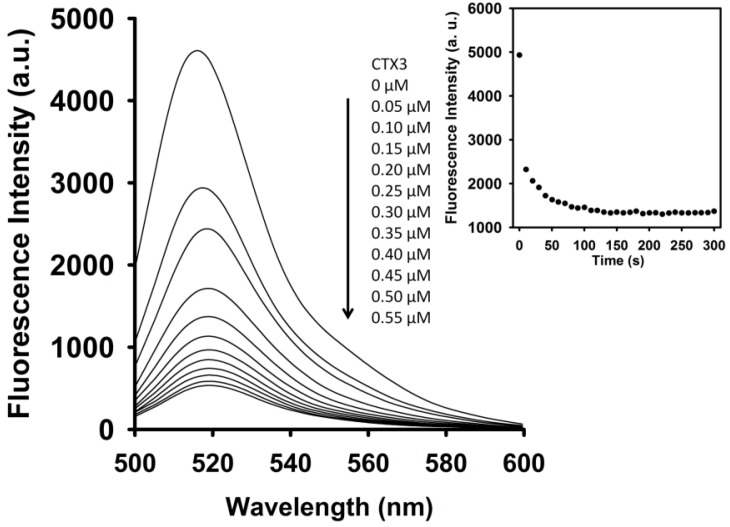
Fluorescence intensity at 520 nm of A_12_-MB-A_12_ was reduced by titrating with CTX3. FAM/DABCYL-labeled A_12_-MB-A_12_ (10 nM) was titrated with indicated CTX3 concentrations. (**Inset**) Time course measurement of FAM intensity (520 nm) of 10 nM A_12_-MB-A_12_ upon the addition of 200 nM CTX3.

As shown in Figure 3, CTX3 induced a decrease in the FAM fluorescence of A_12_-MB-A_12_ in the absence of coralyne in a concentration-dependent manner, and maximal reduction in FAM fluorescence was noted when CTX3 was ≥550 nM. As shown in inset of Figure 3, CTX3-induced fluorescence turn-off required 120 s to achieve maximum reduction in FAM fluorescence. Likewise, titration of A_12_-MB-A_12_ with other CTX isotoxins also resulted in the quenching of FAM fluorescence (Figure S3, Supplementary Materials). To prove that the reduction in fluorescence occurred only upon binding of CTX3 to the A_12_-MB-A_12_, a control experiment was conducted by titrating FAM solution with CTX3. CTX3 did not significantly affect the fluorescence intensity of the FAM solution. Thus, any change in the fluorescence intensity of A_12_-MB-A_12_ was caused by the binding of CTX3. These observations indicated that the binding of CTXs with A_12_-MB-A_12_ might keep DABCYL and FAM in close proximity. The binding affinity of CTX isotoxins for A_12_-MB-A_12_ was calculated using the titration data derived from the change in FAM fluorescence intensity induced by toxin molecules. The dissociation constants (K_d_) of CTX1, CTX2, CTX3, CTX4, CTX5, and CTXn for A_12_-MB-A_12_ were 0.2 μM, 0.5 μM, 0.1 μM, 0.6 μM, 0.1 μM, and 0.1 μM, respectively.

**Figure 4:**
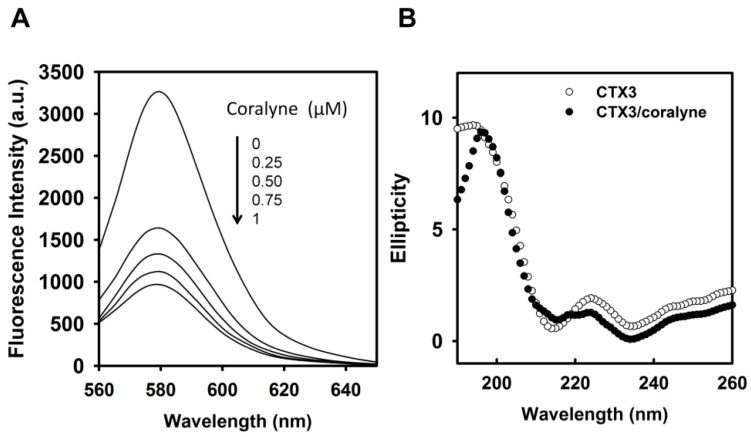
Fluorescence and CD measurements of the interaction of coralyne with CTX3. (**A**) Rhodamine-labeled CTX3 (0.2 μM) was titrated with indicated coralyne concentrations; (**B**) CD spectra of CTX3 and CTX3-coralyne complexes. The used CTX3 and coralyne concentrations were 280 μM and 1400 μM, respectively.

**Figure 5:**
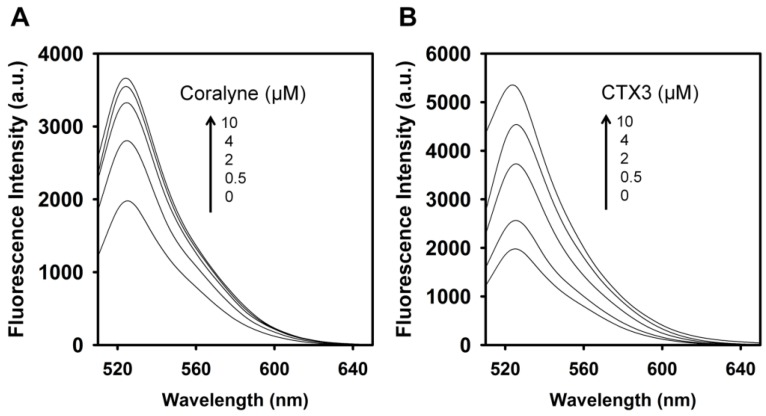
Change in fluorescence spectra of a solution of SG and label-free A_12_-MB-A_12_ after the addition of coralyne or CTX3. Fluorescence spectra of solutions of 160 nM SG and 0.5 μM label-free A_12_-MB-A_12_ in the presence of indicated (**A**) coralyne and (**B**) CTX3 concentrations.

As per the above findings, CTX3 disrupted the coralyne-induced stem-loop MB structure and quenched the FAM fluorescence of A_12_-MB-A_12_ irrespective of coralyne, hence two possibilities could be considered: (1) CTX3 bound to coralyne; and (2) CTX3 induced formation of the stem-loop MB structure. As shown in Figure 4A, coralyne induced a reduction in the fluorescence intensity of rhodamine-labeled CTX3 in a concentration-dependent manner. The affinity of CTX3 with coralyne was calculated from the change in fluorescence intensity. The dissociation constant (K_d_) of CTX3 for coralyne was 0.2 μM. CD spectra also showed that coralyne induced a change in the gross conformation of CTX3 (Figure 4B). These observations indicated the binding of CTX3 with coralyne. Molecular docking analyses suggest the involvement of Lys2, Tyr 11, Thr13, Asp57, and Arg58 of CTX3 in binding with coralyne (Figure S4, Supplementary Materials). To test whether CTX3 induced a stem-loop conformation of MB, SG fluorescence enhancement was used to detect folded MB structure. SG is an organic dye that has weak fluorescence but exhibits a fluorescence enhancement upon binding to double-stranded DNA. Compared with the fluorescence intensity of SG in the presence of coralyne-free A_12_-MB-A_12_, the fluorescence intensity in the presence of coralyne-A_12_-MB-A_12_ complexes was markedly enhanced (Figure 5A). It was consistent with the notion that coralyne induced the hairpin-shaped structure of A_12_-MB-A_12_. Likewise, following the addition of CTX3, an obvious increase in the fluorescence intensity at 525 nm of SG was noted (Figure 5B). Apparently, CTX3 rendered MB to form a folded structure.

**Figure 6:**
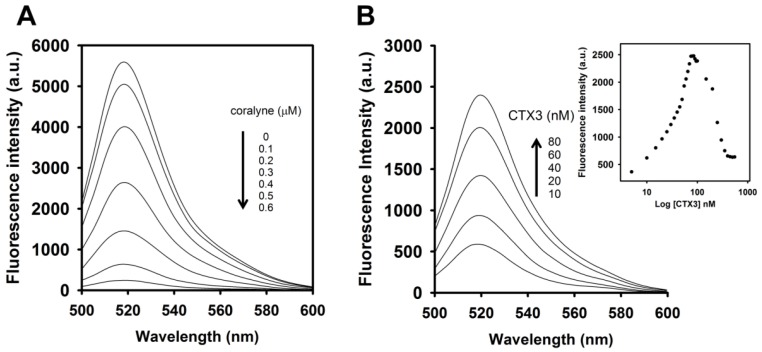
Effect of coralyne and CTX3 on fluorescence intensity at 520 nm of FAM-A_40_-DABCYL and coralyne-FAM-A_40_-DABCYL complexes. (**A**) FAM/DABCYL-labeled A_40_ (10 nM) was titrated with increasing coralyne concentrations as indicated; (**B**) Effect of CTX3 on fluorescence intensity at 520 nm of hairpin-shaped A_40_. Fluorescence spectra of solutions of 10 nM FAM/DABCYL-labeled A_40_ and 0.6 μM coralyne in the presence of indicated CTX3 concentrations. (**Inset**) Effect of CTX3 on fluorescence intensity at 520 nm of a solution containing 10 nM FAM/DABCYL-labeled A_40_ and 0.6 μM coralyne.

**Figure 7:**
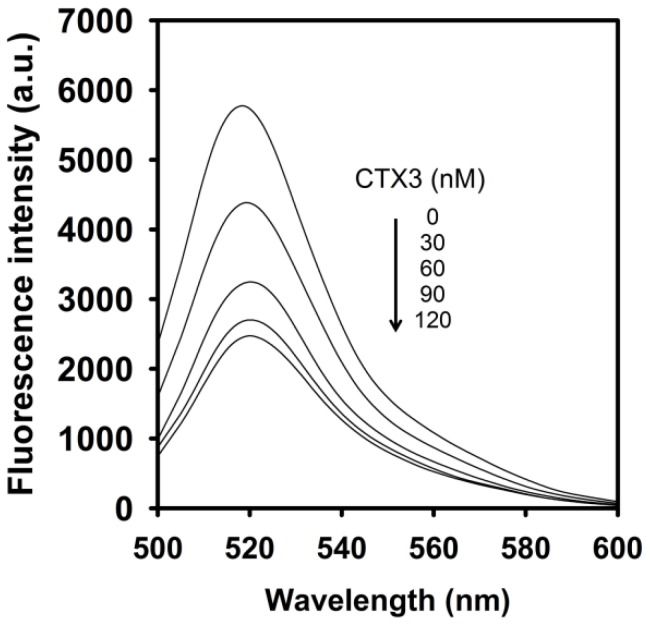
Fluorescence intensity at 520 nm of FAM/DABCYL-labeled A_40_ was reduced by titrating with CTX3. FAM/DABCYL-labeled A_402_ (10 nM) was titrated with indicated CTX3 concentrations.

Previous studies showed that coralyne could induce hairpin structure of poly A_40_ [18]. Thus, the effect of coralyne on fluorescence of FAM-A_40_-DABCYL was analyzed. As shown in Figure 6A, the fluorescence intensity of FAM was reduced by the addition of coralyne. The fluorescence quenching reached a saturation level after addition of 0.6 μM coralyne, indicating that the hairpin structure of A_40_ was more stable at coralyne concentration exceeding 0.6 μM. To assess the concentration-dependent effect of CTX3 on the FAM fluorescence of hairpin-shaped A_40_, a solution containing 10 nM FAM-A_40_-DABCYL and 0.6 μM coralyne was titrated with CTX3. As shown in Figure 6B, maximum restoration of FAM fluorescence was noted with the addition of 80 nM CTX3. Moreover, a notable reduction in the FAM fluorescence of FAM-A_40_-DABCYL was noted when the concentration of added CTX3 was >100 nM (Inset of Figure 6B), and thus titration of coralyne- FAM-A_40_-DABCYL complexes with CTX3 also yielded a bell-shaped curve of FAM fluorescence signal. As shown in Figure 7, CTX3 induced a decrease in the FAM fluorescence of FAM/DABCYL-labeled A_40_ in a concentration-dependent manner, and maximal reduction in FAM fluorescence was noted when CTX3 was ≥120 nM. The binding affinity of CTX3 for A_40_ was calculated using the titration data derived from the change in FAM fluorescence intensity induced by toxin molecules. The dissociation constants (K_d_) of CTX3 for A_40_ was 0.17 μM. These findings supported that CTX3 binds with the poly dA part of MB.

**Figure 8:**
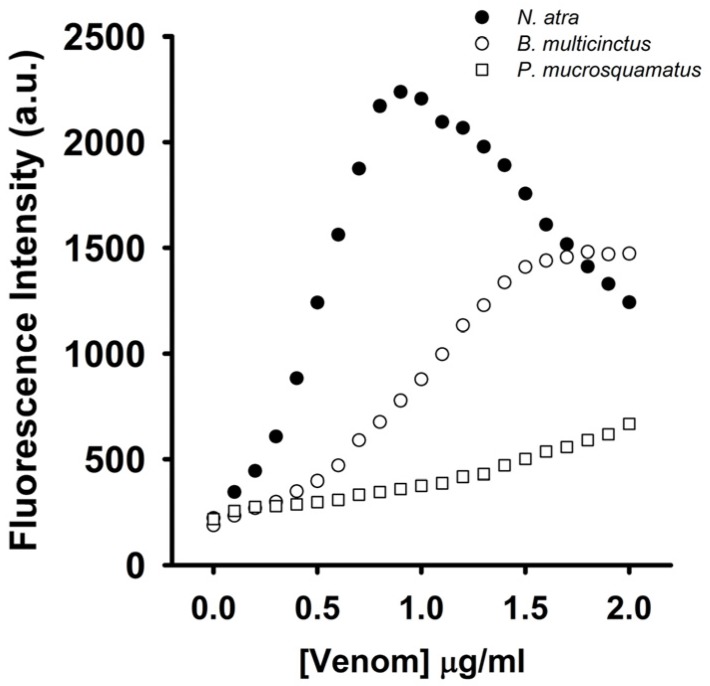
Effect of *N. atra*, *B. multicinctus*, and *P. mucrosquamatus* venoms on fluorescence intensity at 520 nm of a solution containing 10 nM FAM/DABCYL-labeled A_12_-MB-A_12_ and 0.6 μM coralyne. The hairpin-shaped MB was titrated with indicated concentrations of *N. atra*, *B. multicinctus*, and *P. mucrosquamatus* venoms.

To test whether the coralyne-A_12_-MB-A_12_ complexes could specifically detect CTX-containing venom, the effect of *N. atra*, *B. multicinctus*, and *P. mucrosquamatus* venoms on the FAM fluorescence of hairpin-shaped MB was analyzed. To date, no studies indicate that *B. multicinctus* and *P. mucrosquamatus* venoms contain CTXs. As shown in Figure 8, *N. atra* venom maximally restored MB fluorescence signal at concentration of 0.8 μg/mL, and further increase in the concentration of *N. atra* venom caused a reduction in MB fluorescence intensity. Additionally, *P. mucrosquamatus* venom slightly increased the FAM fluorescence of hairpin-shaped MB at the test concentrations. Although *B. multicinctus* venom could notably restore MB fluorescence intensity, *B. multicinctus* venom did not function as *N. atra* venom to yield a bell-shaped concentration-dependent curve of MB fluorescence signal. Moreover, at concentration ≤0.8 μg/mL, *N. atra* venom showed a superior ability to restore the FAM fluorescence of hairpin-shaped MB compared to *B. multicinctus* and *P. mucrosquamatus* venoms. Taken together, these results show that the hairpin-shaped MB can discriminate *N. atra* venom from *P. mucrosquamatus* and *B. multicinctus* venoms. A linear relationship (*R*^2^ = 0.976) between fluorescent quenching and crude *N. atra* venom was obtained over the range of 0–0.8 μg/mL. The limit of detection for *N. atra* venom was determined to be 0.028 μg/mL at a signal-to-noise ratio of 3 (3σ).

**Figure 9:**
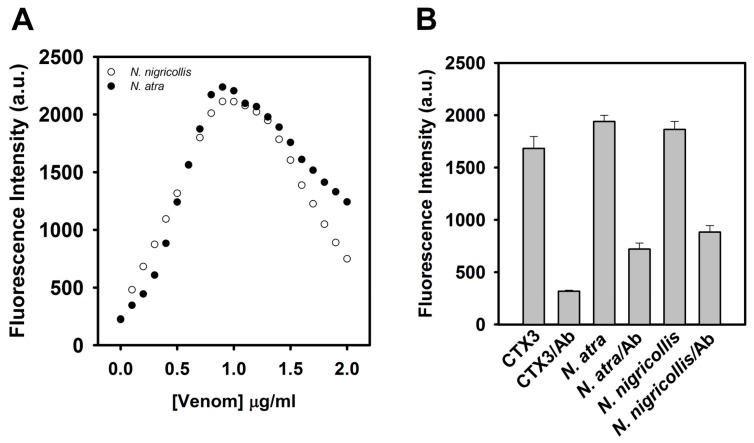
Anti-CTX3 antibodies reduced the ability of CTX3, *N. arta* venom, and *N. nigricollis* venom to recover fluorescence intensity at 520 nm of a solution containing 10 nM FAM/DABCYL-labeled A_12_-MB-A_12_ and 0.6 μM coralyne. (**A**) The hairpin-shaped MB was titrated with indicated concentrations of *N. atra* and *N. nigricollis* venoms; (**B**) CTX3 (40 nM) and crude venoms (0.7 μg) were incubated with anti-CTX3 antibodies (Ab, 40 nM) for 10 min, and then the reaction mixtures were added into the solution of hairpin-shaped MB.

Given that the tested CTX isotoxins caused a recovery of FAM fluorescence of hairpin-shaped MB, it was likely that snake venom containing CTXs might restore FAM fluorescence. Previous studies showed that *N. nigricollis* venom contained CTX isotoxins [19]. As shown in Figure 9A, *N. nigricollis* venom functioned as *N. atra* venom to yield a bell-shaped concentration-dependent curve of MB fluorescence signal. Figure 9B shows that preincubation with anti-*N. atra* CTX3 antibodies reduced the ability of CTX3, *N. atra* venom and *N. nigricollis* venom to restore FAM fluorescence of hairpin-shaped MB. These findings indicated that the hairpin-shaped MB could detect crude venoms containing CTXs, and suggested that the change of MB fluorescence signal could not differentiate the venom of *N. atra* from the venoms of other *Naja* species.

**Figure 10:**
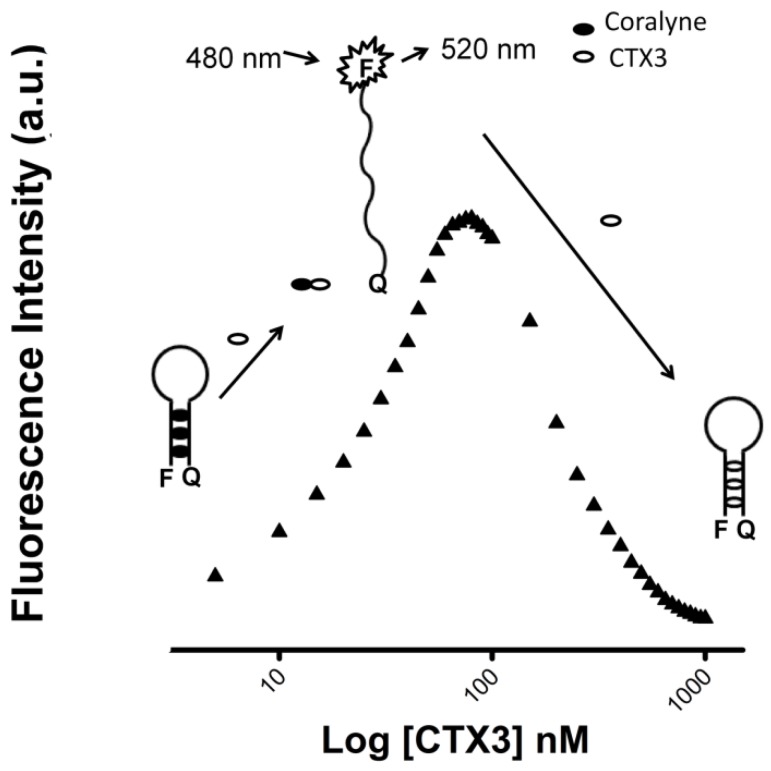
Schematic drawing showing CTX3-induced bell-shaped concentration-response curves with FAM fluorescence of A_12_-MB-A_12_-coralyne complexes.

Previous studies revealed that CTXs could bind with aptamers against α-bungarotoxin [20]. Aptamers are synthetic oligonucleotides, such as RNA and single-stranded DNA, that can bind to their targets due to their specific secondary or tertiary structures [21,22]. Therefore, aptamer-binding by proteins is largely determined by how well the molecules fit into the cavities of the target proteins [23,24]. Chen et al. [20] propose that the binding of CTXs to aptamers against α-bungarotoxin is due to the structural complementarity. Noticeably, CTXs and α-bungarotoxin show similar binding affinity for aptamers, and thus the aptamers are unable to discriminate CTXs from α-bungarotoxin. Obviously, compared to aptamers against α-bungarotoxin, the coralyne-MB complexes platform specifically detects CTXs. In the present study, our data revealed that CTX3 could bind with coralyne and A_12_-MB-A_12_. At concentrations ≤80 nM, CTX3 converts MB from a stem-loop structure to a random-coil structure via binding with coralyne in a concentration-dependent manner; when toxin concentration further increased, CTX3 causes the random-coil MB structure to become a folded structure irrespective of coralyne (Figure 10). Thus, the FAM fluorescent signal of A_12_-MB-A_12_-coralyne complexes yielded a bell-shaped curve as a function of CTX3 concentration. Likewise, the FAM fluorescence signal of A_12_-MB-A_12_-coralyne complexes also yielded bell-shaped curves in response to other CTX isotoxins (Figure S3, Supplementary Materials). Given that the binding affinity of CTX3 for coralyne (K_d_, 0.2 μM) was greater than that of A_12_-MB-A_12_ for coralyne (K_d_, 0.5 μM), it is conceivable that CTX3 can competitively remove coralyne from the MB. Noticeably, Figure 3 shows that 550 nM CTX3 maximally induces fluorescence quenching of MB, while 1000 nM CTX3 is required for maximally quenching MB fluorescence in the presence of 0.6 μM coralyne (Figure 2B). These observations suggest that the interaction between coralyne and CTX3 attenuates the binding capability of CTX3 with A_12_-MB-A_12_. However, the binding affinity of CTX3 for coralyne (K_d_, 0.2 μM) was lower than that of CTX3 for A_12_-MB-A_12_ (K_d_, 0.1 μM). Thus, increasing CTX3 concentration results in the folded MB structure in the presence of coralyne. Our data show that CTX3 causes a ‘turn-on’ fluorescence signal of hairpin-shaped MB at a concentration ≤80 nM in a concentration-dependent manner, while cobrotoxin and α-bungarotoxin marginally affects the FAM fluorescence of hairpin-shaped MB at the test concentrations (Figure 2B). These observations suggest that the coralyne-induced hairpin conformation of A_12_-MB-A_12_ can discriminate CTX isotoxins from α-neurotoxin. When CTX3 concentration was ≤80 nM, CTX3 concentration-dependently disrupted stem-loop structure of MB via removal of coralyne from MB. Thus, CTX3 concentration-dependently restores FAM fluorescence of MB. Increase in CTX3 concentration further caused the formation of folded MB structure, leading to quenched FAM fluorescence by DABCYL. Thus, a bell-shaped curve was noted with FAM fluorescence intensity of coralyne-A_12_-MB-A_12_ complexes in response to titration with CTX3. Hung et al. [25] reported that the serum venom levels of *N. atra* snakebite patients are 228–1270 ng/mL after 2 h snakebite. Our data show that the detection limit for crude *N. naja atra* venom is 28 ng/mL. Thus, human serum samples spiked with *N. atra*, *B. multicinctus*, and *P. mucrosquamatus* venoms were used to test the utility of the MB for detecting CTXs in the presence of serum proteins. Unfortunately, serum proteins alone caused a notable recovery of hairpin-shaped MB fluorescence signal, and hence the effect *N. atra*, *B. multicinctus*, and *P. mucrosquamatus* venoms on hairpin-shaped MB fluorescence signal were unable to be detected (data not shown). Evidently, the MB probe could not be free from matrix effect of the plasma sample. Thus, the potential applicability of the method for detecting serum snake venom from snakebite patients needs some alterations. Previous studies revealed that human serum albumin and bovine serum albumin could bind with coralyne [26]. Removal of the serum proteins might be the required step before the use of MB probe for detecting CTXs in human serum samples. On the other hand, the detection sensitivity of ELISA for crude *N. atra* venom has been reported to be 1 ng/mL [25]. It is obvious that the sensitivity of MB should be further improved.

In summary, the present findings suggest the utility of an adenosine-based MB for rapidly sensing CTXs. The detected sensitivity can be achieved at nanomolar level, suggesting that this is a sensitive method for detecting CTXs. Moreover, the fact that the FAM fluorescence of A_12_-MB-A_12_-coralyne complexes yielded bell-shaped curves in response to CTXs indicates that this specific character could also be used for detecting CTXs.

## 3. Materials and Methods

Crude venoms of *Naja atra* (Taiwan cobra)*, Bungarus multicinctus* (Taiwan banded krait), and *Protobothrops mucrosquamatus* (Taiwan habu) were milked from the venom glands of at least 40 adult snakes in Taiwan. The crude venoms were pooled together and stored at −20 °C after lyophilization. Cobrotoxin and CTX isotoxins (CTX1, CTX2, CTX3, CTX4, CTX5, and CTXn) were isolated from the venom of *N. atra* according to the procedure described in Lin et al. [27]. Purification of α-bungarotoxin from *B. multicinctus* venom was carried out according to the procedure described in Chang et al. [13]. Moreover, the pooled crude venoms were used for studying their effect on the fluorescent signal of coralyne-MB complexes. The chromatographic profiles for separation of CTX3, cobrotoxin, and α-bungarotoxin were shown in Figure S5A (Supplementary Materials), and the purity of CTXs, cobrotoxin, and α-bungarotoxin was verified using MALDI-TOF analyses (Figure S5B, Supplementary Materials). Rabbit anti-*N. atra* CTX3 sera were prepared in our laboratory [28], and anti-CTX3 antibodies were further purified using a CTX3-Sepharose column that was prepared by covalent coupling of CTX3 to CNBr-activated Sepharose 4B column. SYBR Green I (SG) was purchased from Molecular Probe Inc. (Eugene, OR, USA). Calcein, carboxyfluorescein (FAM), coralyne sulfoacetate, HEPES, crude venom of *N. nigricollis*, and rhodamine B isothiocyanate were purchased from Sigma-Aldrich Inc. (St. Louis, MO, USA); and 5′-FAM/3′-DABCYL-labeled A_12_-CATCATAGTCGAGTGTCCAGGG-A_12_ DNA MB, 5′-FAM/3′-DABCYL-labeled A_40_, and label-free A_12_-CATCATAGTCGAGTGTCCAGGG-A_12_ were synthesized from Neogene Biomedicals Corporation (Taipei, Taiwan). CNBr-activated Sepharose 4B was obtained from GH Healthcare Bio-Sciences Corp. (Piscataway, NJ, USA). Unless otherwise specified, all other reagents were analytical grade.

### 3.1. Circular Dichroism (CD) Measurement

CD spectra were obtained on a Jasco J-810 spectropolarimeter (JASCO corporation, Tokyo, Japan) with a cell path-length of 0.5 mm. The CD spectra were measured from 260 nm to 190 nm, and CD spectra were obtained by averaging the signals of five scans.

### 3.2. Competitive Binding of CTXs and Coralyne to MB

All samples were prepared in solution containing 10 mM HEPES (pH 7.3). The FAM/DABCYL-labeled A_12_-MB-A_12_ (10 nM) were titrated with small aliquots of CTXs in the presence of 0.6 μM coralyne. Total dilution never exceeded 10% and the relative fluorescence values were uniformly corrected for dilution. Titration of CTXs was stopped when fluorescence intensity of MB no longer changing. The fluorescence spectra were measured on a Hitachi F-4500 Fluorescence spectrophotometer (Hitachi High-Technologies Corporation, Tokyo, Japan) with excitation wavelength at 480 nm. To measurement the dissociation constant of CTXs with MB, FAM/DABCYL-labeled A_12_-MB-A_12_ (10 nM) were titrated with small aliquots of CTXs in the absence of coralyne. A plot of the 1/(Fo-F) versus 1/[Toxin] gives lines with a slope corresponding to the dissociation constant of MB-toxin complexes. Fo and F are fluorescence intensity in the absence or presence of toxins. To study the effect of anti-CTX3 antibodies on the binding of CTX3 and crude venom to hairpin-shaped MB, CTX3, and crude venom were preincubated with anti-CTX3 antibodies for 10 min. The FAM fluorescence of MB was measured after addition of the reaction mixtures.

### 3.3. Binding of Rhodamine-Labeled CTX3 with Coralyne

Rhodamine-labeled CTX3 was prepared according to the procedure described in Chen et al. [29]. Rhodamine-labeled CTX3 (0.2 μM) was titrated with increasing concentrations of coralyne until maximal changes in fluorescence intensity of rhodamine-labeled CTX3 was achieved. The binding was monitored at excitation wavelength and emission wavelength at 550 and 580 nm, respectively. A plot of the 1/(Fo-F) versus 1/[coralyne] gives lines with a slope corresponding to the dissociation constant of CTX3-coralyne complexes. Fo and F are fluorescence intensity in the absence or presence of coralyne.

### 3.4. Measurement of Coralyne- and CTX3-Induced Folded Structure of Label-Free MB Using SG Fluorescence Enhancement

A stock solution of SG (12.3 μM) was prepared according to the procedure described in Lin and Tseng [18]. SG (160 nM) and label-free MB (0.5 μM) were incubated in 10 mM HEPES (pH 7.3) for 40 min until the SG fluorescence intensity was no more changed. Then the solution containing SG and label-free MB was titrated with coralyne and CTX, respectively. The fluorescence spectra were measured on a Hitachi F-4500 Fluorescence spectrophotometer with the excitation wavelength at 494 nm.

### 3.5. Molecular Docking

Molecular docking of the binding of coralyne with CTX3 was conducted using iGEMDOCK v2.1 (BioXGEM Lab., Hsinchu, Taiwan). We used its molecular docking platform to dock the coralyne to CTX3 with a population size of 300, a number of generations of 80 and the number of solutions set to 100. The 100 docking scores were then used for statistical analysis to evaluate the binding model of CTX3 with coralyne.

## Supplementary Materials

The following are available online at www.mdpi.com/2072-6651/9/1/24/s1, Figure S1: Time course measurement of FAM intensity (520 nm) of hairpin-shaped MB upon the addition of 100 nM CTX3; Figure S2: Effect of CTX isotoxins on fluorescence intensity at 520 nm of a solution containing 10 nM FAM/DABCYL-labeled A_12_-MB-A_12_ and 0.6 μM coralyne; Figure S3: Fluorescence intensity at 520 nm of A_12_-MB-A_12_ was reduced by titrating with CTX isotoxins; Figure S4: Molecular docking showing the binding of CTX3 with coralyne; Figure S5: Chromatographic separation and MALDI-TOF analyses of CTX3, cobrotoxin, and α-bungarotoxin.

## Abbreviations

The following abbreviations are used in this manuscript:

CTX	Cardiotoxin
DABCYL	4-([4-(dimethylamino)phenyl]azo)-benzoic acid
FAM	Carboxyfluorescein
