# Supplementary material for: Detection of Naja atra Cardiotoxin Using Adenosine-Based Molecular Beacon
Source: Toxins (Basel). 2017 Jan 7;9(1):24. doi: 10.3390/toxins9010024 (PMC5308256; doi:10.3390/toxins9010024)
Supplement: Supplementary file 1 [file toxins-09-00024-s001.pdf]

Supplementary Materials: Detection of *Naja atra* Cardiotoxin Using Adenosine-Based Molecular Beacon

Yi-Jun Shi, Ying-Jung Chen, Wan-Ping Hu and Long-Sen Chang

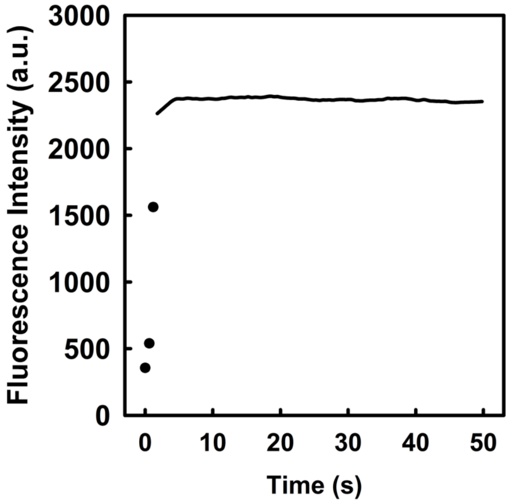

Figure S1. Time course measurement of FAM intensity (520 nm) of hairpin-shaped MB upon the addition of 100 nM CTX3. The solution containing 10 nM FAM/DABCYL-labeled A_{12}-MB-A_{12} and 0.6 μM coralyne was incubated with 80 nM CTX3 as indicated time periods.

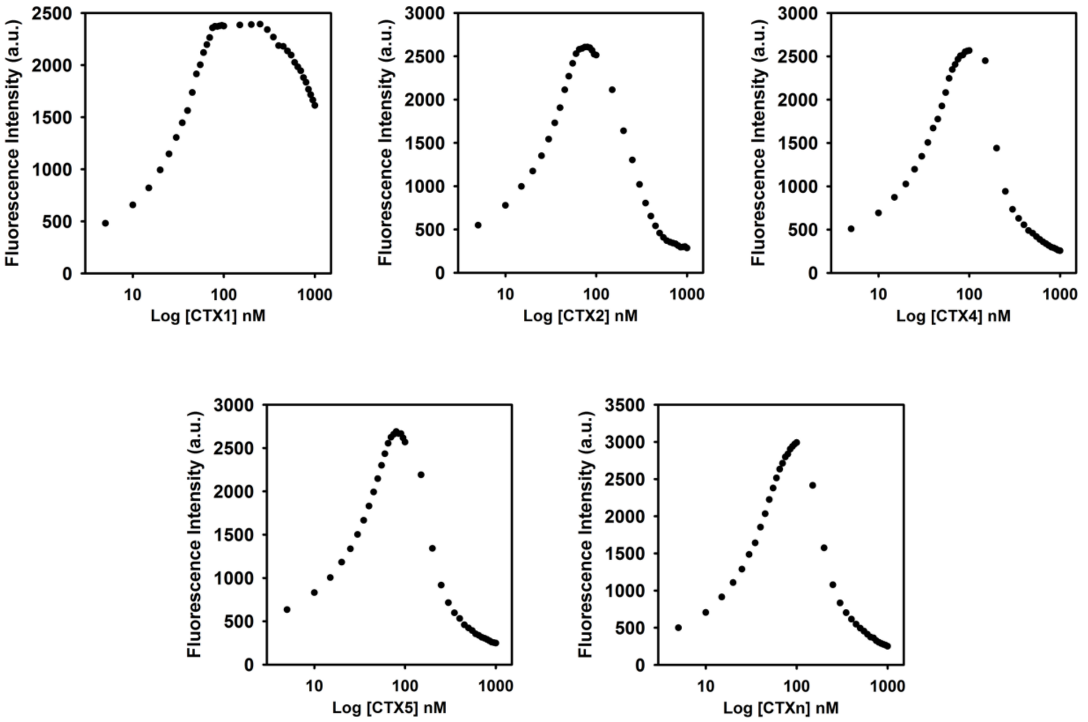

Figure S2. Effect of CTX isotoxins on fluorescence intensity at 520 nm of a solution containing 10 nM FAM/DABCYL-labeled A_{12}-MB-A_{12} and 0.6 μM coralyne. The hairpin-shaped MB was titrated with indicated concentration of CTXs.

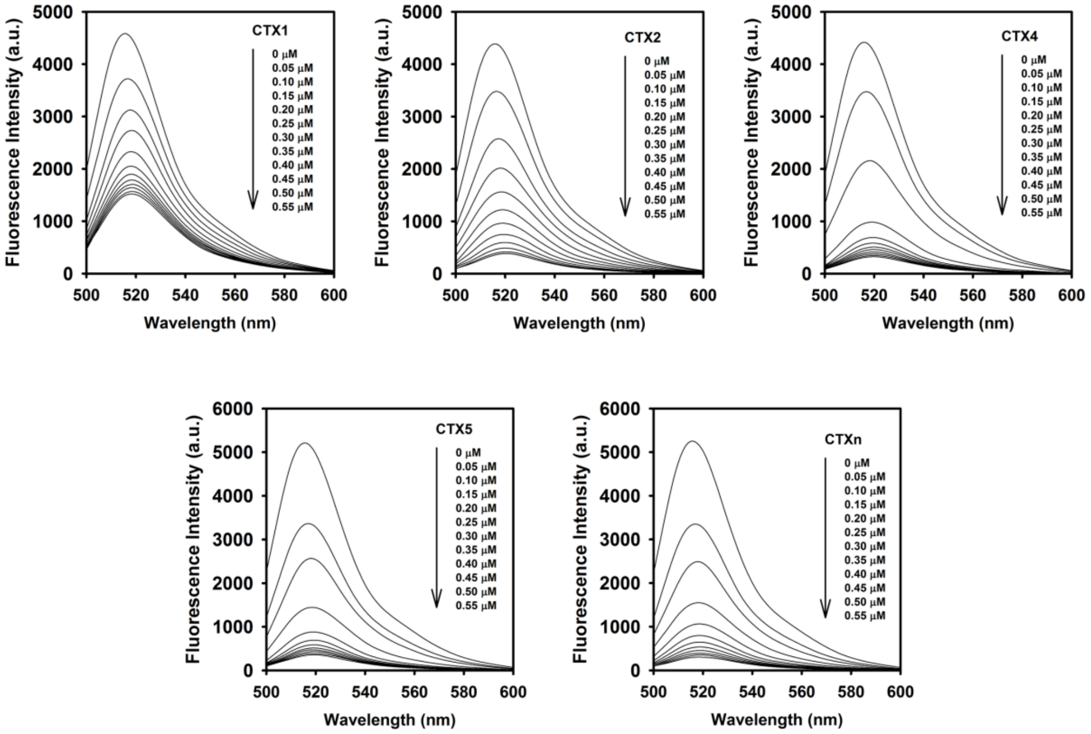

Figure S3. Fluorescence intensity at 520 nm of A_{12}-MB-A_{12} was reduced by titrating with CTX isotoxins. FAM/DABCYL-labeled A_{12}-MB-A_{12} (10 nM) was titrated with indicated concentrations of CTXs.

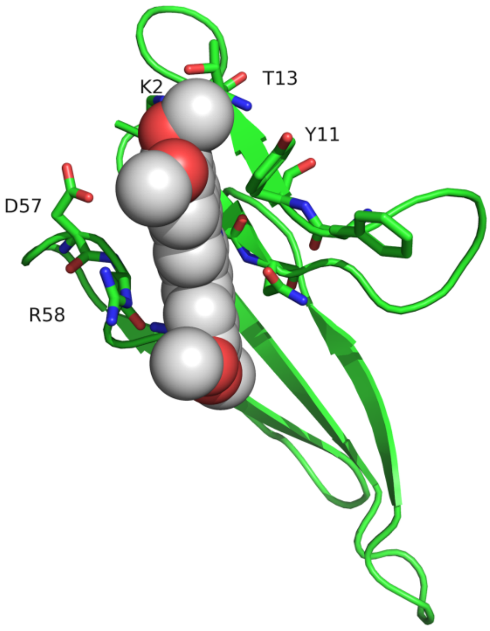

Figure S4. Molecular model showing the binding of coralyne with CTX3.

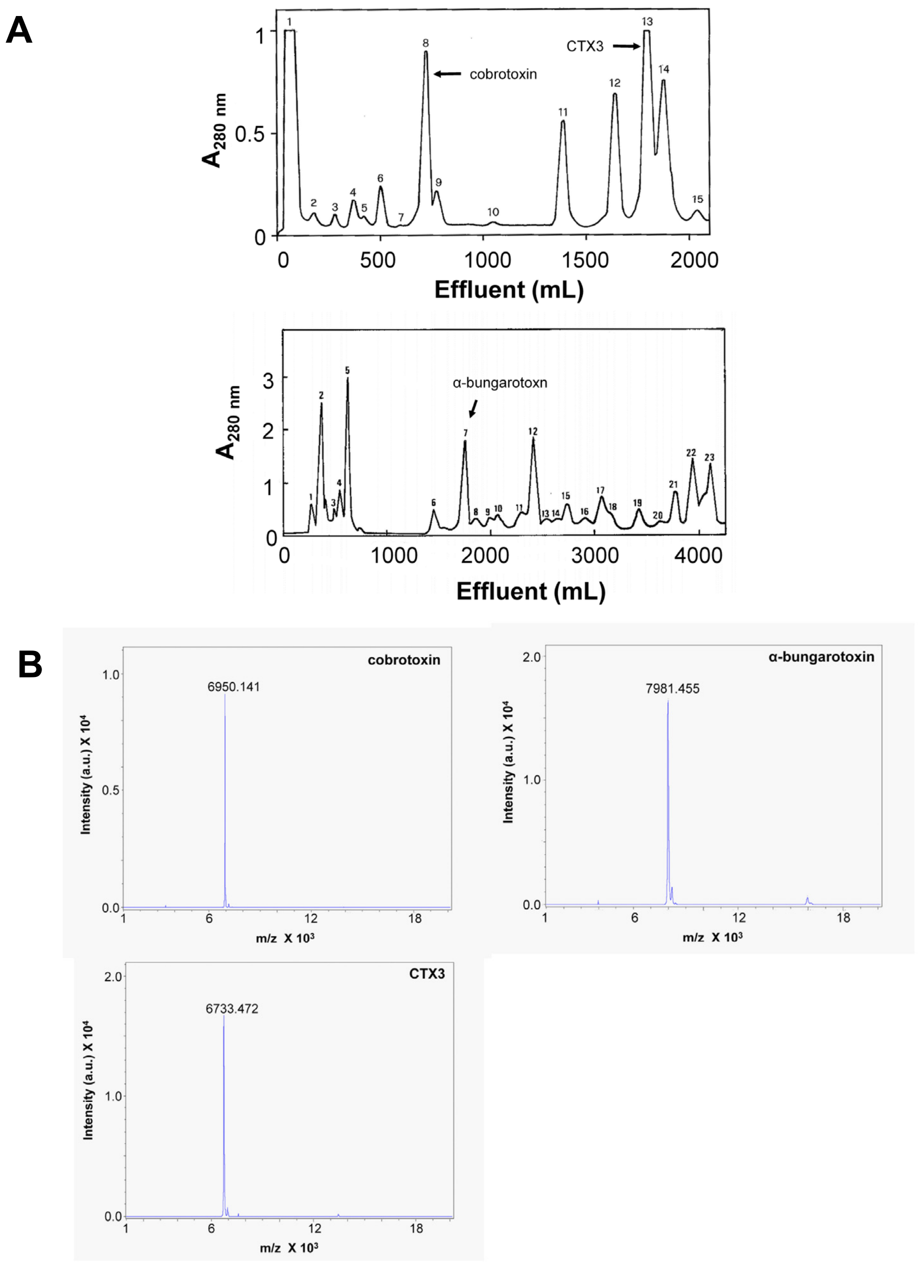

Figure S5. Chromatographic separation and MALDI-TOF analyses of CTX3, cobrotoxin, and α-bungarotoxin. (**A**) Separation of CTX3, cobrotoxin, and α-bungarotoxin from *N. atra* and *Bungarus multicinctus* crude venoms were conducted essentially according to the same manner described in [1,2]; (**B**) MALDI-TOF analyses of CTXs, cobrotoxin, and α-bungarotoxin.

References

1. Chang, L.S.; Lin, S.K.; Huang, H.B.; Hsiao, M. Genetic organization of α-bungarotoxins from *Bungarus multicinctus* (Taiwan banded krait): Evidence showing that the production of α-bungarotoxin isotoxins is not derived from edited mRNAs. *Nucleic Acids Res.* **1999**, *27*, 3970–3975.
2. Lin, S.R.; Chang, L.S.; Chang, K.L. Separation and structure-function studies of Taiwan cobra cardiotoxins. *J. Protein Chem.* **2002**, *21*, 81–86.

© 2017 by the authors. Submittedfor possible open access publication under the terms and conditions of the Creative Commons Attribution (CC-BY) license (http://creativecommons.org/licenses/by/4.0/).
